# The Relationship between Childhood Maltreatment and Non-Suicidal Self-Injury: A Systematic Review

**DOI:** 10.3389/fpsyt.2017.00149

**Published:** 2017-08-24

**Authors:** Gianluca Serafini, Giovanna Canepa, Giulia Adavastro, Jacopo Nebbia, Martino Belvederi Murri, Denise Erbuto, Benedetta Pocai, Andrea Fiorillo, Maurizio Pompili, Eirini Flouri, Mario Amore

**Affiliations:** ^1^Department of Neuroscience, Rehabilitation, Ophthalmology, Genetics and Maternal and Child Health, Section of Psychiatry, University of Genoa, Genoa, Italy; ^2^Department of Neurosciences, Mental Health and Sensory Organs, Suicide Prevention Center, Sant’Andrea Hospital, Sapienza University of Rome, Rome, Italy; ^3^Department of Psychiatry, University of Naples SUN, Naples, Italy; ^4^Department of Psychology and Human Development, UCL Institute of Education, University College London, London, United Kingdom

**Keywords:** childhood maltreatment, physical/sexual abuse, emotional neglect, non-suicidal self-injury, suicidal behaviors

## Abstract

**Introduction:**

Childhood maltreatment (CM) has been associated with an increased risk of non-suicidal self-injury (NSSI) and suicidal behaviors. However, the exact nature of the association between CM and NSSI is currently unclear. The present review aimed to systematically investigate the association between CM and NSSI in adolescence and early adulthood.

**Methods:**

A systematic search of four major electronic databases covering both medical and social science research (PubMed, Scopus, Science Direct, and PsycINFO) was conducted.

**Results:**

Overall, 20 cross-sectional studies including a total of 22,517 individuals, 3 longitudinal follow-up studies including 1,728 individuals, and 3 retrospective studies including 62,089 individuals were selected. It appears that CM is a significant risk factor for both NSSI and suicide attempts. The increased vulnerability to NSSI seems to be related to experiences of CM, particularly sexual abuse. Gender differences were also found. Generally, when compared to males, females who experienced CM seem to be more vulnerable to presenting with NSSI and suicidal behaviors.

**Conclusion:**

There is a positive association between CM and NSSI. The importance of early detection and risk reduction of self-injurious behavior for adolescents is discussed.

## Introduction

Non-suicidal self-injury (NSSI) may be defined as the direct, deliberate destruction of one’s own body without suicidal intent (1), and it has been described as a serious public health concern for adolescents and young adults (2). Using data from community and psychiatric samples, Grandclerc and colleagues (3) reported that the prevalence of adolescent NSSI is between 10 and 35%. Individuals experiencing NSSI thoughts and those engaging in NSSI behaviors report poorer social relationships and greater psychosocial impairment, compared with their counterparts (4). Importantly, NSSI is associated with an increased risk of psychopathological conditions (5, 6). In adolescence, a significant percentage of both community self-injurers and inpatients reporting NSSI attempt suicide (7, 8).

Self-harming behavior rates in those with a history of childhood maltreatment (CM) are higher than in the general population (9), in line with much evidence showing that the psychopathological potential and consequences of CM are quite broad (10). CM can be subdivided into the following domains (11): (1) physical abuse, i.e., deliberate physical harm; (2) emotional abuse such as verbal aggression significantly affecting the child’s wellbeing, or behaviors that may humiliate, embarrass, or seriously threaten the child; (3) sexual abuse, i.e., any type of sexual contact/behavior between a child and an adult; (4) emotional neglect, i.e., the failure to satisfy vital emotional and psychological needs such as love and support; and (5) physical neglect, i.e., the failure to meet basic physical needs such as food and shelter.

Small-to-medium associations have been reported between CM and the presence of a recent history of NSSI (12). Maltreatment has also been associated with an increased risk of adolescent suicidal behaviors, particularly in females (13). Some recent evidence suggests specificity in both CM and injury behaviors in adolescence (14), with a history of physical/sexual abuse being associated with an increased risk of suicide attempts and a history of neglect with an increased risk of NSSI. However, the exact nature of the association between CM and NSSI in adolescence and early adulthood is currently unclear (15). The consequences for prolonged, chronic engagement in NSSI, or for intermittent versus repetitive NSSI represent additional aspects that need to be explored.

Thus, given this background, the present review aims to systematically investigate the association between CM and NSSI in adolescence and early adulthood. We hypothesized that CM is associated with an increased vulnerability to NSSI, although we postulated that this association may be moderated or mediated by other factors, not necessarily linked to the characteristics of CM. Therefore, the present review seeks to add to the literature on the link between CM and NSSI, while providing a more in depth analysis of the paths leading to NSSI following an experience of CM.

## Methods

### Eligibility Criteria

We adopted the “Preferred Reporting Items for Systematic Reviews and Meta-Analyses” guidelines (16). We included studies that explicitly mentioned the association between child maltreatment (OR child abuse OR child neglect) AND NSSI in childhood, adolescence, or adulthood. When a title or abstract seemed to describe a study suitable for inclusion, the full-text article was obtained and carefully examined to evaluate its relevance for our work. Our exclusion criteria were as follows: (1) studies published before 1980; (2) studies without abstracts or with abstracts that did not explicitly mention the association between CM and NSSI or self-harm in childhood, adolescence, or adulthood; (3) studies that were not published in English; (4) systematic reviews or meta-analytic studies on the topic; and (5) studies in which maltreatment was perpetrated by other children.

### Information Sources

We conducted a systematic search of four major electronic databases including medical and social science studies (PubMed, Scopus, Science Direct, and PsycINFO) for titles and abstracts relevant to our research questions. We also examined the bibliographies of the retrieved articles for additional papers that might be relevant. Overall, the papers we examined covered the period between 1996 and 2016.

### Search Terms

The following search query was used in all databases: “child maltreatment” AND “non-suicidal self-injury” OR “child abuse” AND “non-suicidal self-injury” OR “child neglect” AND “non-suicidal self-injury.”

### Selection of Studies

Articles were screened and selected in a two-step process to minimize bias. First, three independent researchers (Giovanna Canepa, Giulia Adavastro, and Jacopo Nebbia) conducted the literature search. Any discrepancies between the three reviewers who examined the studies independently for possible inclusion were resolved by consultation with the senior reviewers (Gianluca Serafini and Mario Amore). In the second phase, full-text articles that met our inclusion criteria were retrieved and independently reviewed by Gianluca Serafini and Mario Amore, who discussed the design and characteristics of the studies to decide whether they could be included in the review. If there were doubts about a particular study, then that study was put on the list of those awaiting assessment, pending acquisition of more information, and was then carefully reanalyzed for possible inclusion. Any disagreements in this step were solved by discussion between reviewers.

### Data Collection Process

Giovanna Canepa, Giulia Adavastro, and Jacopo Nebbia extracted the following data elements from the 37 studies screened for this review (see “Study Sample” below): author/s and publication year, presence/absence of control group, psychiatric diagnosis, study design, sample size, physical or biological assessment, psychometric instruments, sample characteristics, limitations, and main conclusions (for more details, see Table 1). Reviewers acquired the full-text of all 37 articles.

**Table 1:** Summary information about all the studies on the association between CM and NSSI included in this review.

Reference	Study design	Sample size	CM	Limitations/shortcomings	Main findings	Quality score	Quality differentiation
Kaplan et al. (33)	Longitudinal study	58 BPD female youths with (*N* = 29) and without (*N* = 29) a history of child abuse	Separation, physical neglect, emotional, physical and sexual abuse, and witnessing violence	The small sample size. The inclusion of only female youths with BPD. Patients were enrolled exclusively from an intensive dialectical behavior therapy program. The follow-up period was short. NSSI was investigated only during the previous year	The abused group reported greater past NSSI compared with controls. No differences in the follow-up period were found. Co-occurrence of physical and sexual abuse was associated with greater past NSSI and suicidality compared to “no history of child abuse” or “experience of sexual abuse only”	I = 1; II = 1; III = 1; IV = 2; V = 2; VI = 1; VII = 0. Total score = 8	Moderate
Stewart et al. (2)	Cross-sectional study	397 adolescent self-injurer inpatients: non-ideators (*n* = 96); suicide ideators (*n* = 149); suicide attempters (*n* = 152)	Physical and sexual abuse	The sample was mostly composed of female adolescents (*N* = 319). NSSI was investigated only during the previous year. Participants were recruited from an acute residential treatment program	Suicide attempters were more likely to use NSSI methods than non-ideators and ideators. Attempters used more severe NSSI methods than non-attempters. Rates of physical abuse differed significantly across groups, with attempters reporting more physical abuse than non-ideators	I = 2; II = 2; III = 0; IV = 2; V = 2; VI = 0; VII = 2. Total score = 10	Good
Reichl et al. (30)	Cross-sectional study	26 adolescent inpatients engaging in NSSI and 26 age- and gender-matched HC	Antipathy, neglect, physical, psychological, and sexual abuse	The small sample size. The sample included mostly female adolescents (*N* = 24). NSSI was explored only during the previous year	Adolescents engaging in NSSI showed significantly higher cortisol awakening responses compared to HC. In the presence of child abuse, HC exhibited flattened diurnal cortisol slopes while those engaging in NSSI exhibited significantly steeper slopes	I = 1; II = 1; III = 0; IV = 2; V = 2; VI = 0; VII = 0. Total score = 6	Moderate
Guvendeger Doksat et al. (14)	Retrospective study	2,518 children and adolescents who were admitted to a general psychiatric hospital: 1,304 reported NSSI	Physical and sexual abuse, neglect	No differentiation between children and adolescents. No utilization of standardized tests for abuse and NSSI. Low number of females. Participants were recruited from a general psychiatric hospital	A positive history of physical and sexual abuse increased the risk of suicide attempts; a history of neglect increased the risk of NSSI	I = 2; II = 0; III = 0; IV = 0; V = 0; VI = 0; VII = 0. Total score = 2	Low
Garisch and Wilson (6)	Longitudinal study	1,162 adolescent students	Physical and sexual abuse	Utilization of screening instruments to evaluate physical and sexual abuse; participants were exclusively students	NSSI was associated with abuse history	I = 2; II = 0; III = 2; IV = 0; V = 1; VI = 0; VII = 0. Total score = 5	Moderate
Glassman et al. (12)	Cross-sectional study	86 adolescents	Physical, sexual, and emotional abuse, physical and emotional neglect	The sample was predominantly female (*N* = 69)	There were significant, small-to-medium associations between specific forms of CM and presence of a recent history of NSSI. Emotional and sexual abuse had the strongest relation with NSSI	I = 1; II = 0; III = 0; IV = 2; V = 2; VI = 0; VII = 0. Total score = 5	Moderate
Isohookana et al. (13)	Longitudinal study	508 adolescent inpatients	Witnessing domestic violence, physical and sexual abuse	Psychometric instruments evaluating NSSI and CM were not specific; participants were recruited from a general psychiatric hospital	Among girls, experience of sexual abuse significantly increased the risk of NSSI. Maltreatment in general was also associated with an increased risk of NSSI in girls	I = 2; II = 0; III = 2; IV = 1; V = 1; VI = 0; VII = 0. Total score = 6	Moderate
Johnstone et al. (5)	Cross-sectional study	372 MDD adult outpatients	Level of care received from parents, psychological, physical and sexual abuse	The absence of standardized instruments to investigate NSSI. Patients had all MDD	Low paternal care was associated with NSSI. Abuse was not significantly associated with NSSI	I = 2; II = 0; III = 0; IV = 2; V = 0; VI = 0; VII = 0. Total score = 4	Moderate
Kara et al. (24)	Cross-sectional study	295 children and adolescents involved in the justice system	Physical, emotional, and sexual abuse	No differentiation between children and adolescents. Instruments evaluating NSSI and CA were not specific; the sample included predominantly males (*N* = 223) and was drawn from a forensic adolescent population	Sexual abuse was more common in the NSSI group	I = 2; II = 0; III = 0; IV = 1; V = 1; VI = 0; VII = 0. Total score = 4	Moderate
Martin et al. (4)	Cross-sectional study	1,296 adolescent and young adult students: no NSSI control group (*N* = 1,080); NSSI thoughts only group (*N* = 126); NSSI action group (*N* = 90)	Level of care received from parents, relationships with parents and peers, sexual and physical abuse	The sample was predominantly female (*N* = 967). Instruments evaluating CM were not specific. NSSI was only asked about the last 6 months	Individuals engaging in NSSI actions reported poorer relationships with parents and more physical abuse than the no NSSI group	I = 2; II = 2; III = 0; IV = 1; V = 2; VI = 0; VII = 0. Total score = 7	Moderate
Martin et al. (19)	Cross-sectional study	957 university students, of whom 86 engaged in NSSI	Psychological, sexual, and physical abuse/neglect	The sample was predominantly female (*N* = 747). Participants were all university students	Perceived parent–child relational trauma was uniquely linked with NSSI behavior after accounting for perceived CM. Perceived maltreatment by the father was uniquely related to NSSI addictive characteristics	I = 2; II = 0; III = 0; IV = 2; V = 2; VI = 0; VII = 0. Total score = 6	Moderate
Muehlenkamp et al. (31)	Cross-sectional study	422 young adult females admitted to an inpatient treatment unit for eating disorders	Emotional neglect and abuse, physical and sexual abuse	The sample included only females; participants were all admitted to an inpatient treatment unit for eating disorders	Childhood traumatic experiences appeared to have an indirect association with NSSI, *via* low self-esteem, psychopathology, body dissatisfaction, and dissociation	I = 1; II = 0; III = 0; IV = 2; V = 2; VI = 0; VII = 0. Total score = 5	Moderate
Shenk et al. (32)	Cross-sectional study	211 adolescent females, of whom 129 were maltreated and 82 were not	Physical neglect, physical and sexual abuse	The sample included only females	There was a significant association between CM and self-injury. Post-traumatic stress symptoms mediated the relationship between CM and self-injury	I = 1; II = 1; III = 0; IV = 0; V = 1; VI = 0; VII = 0. Total score = 3	Low
Stewart et al. (26)	Cross-sectional study	2,013 adolescents who received mental health services, of whom 407 engaged in NSSI	Emotional, sexual, and physical abuse	The sample included patients with different psychiatric conditions. NSSI was only asked about the previous year	The experience of sexual abuse was associated with NSSI	I = 2; II = 0; III = 0; IV = 0; V = 0; VI = 0; VII = 0. Total score = 2	Low
Stinson et al. (27)	Cross-sectional study	381 forensic mental health inpatients, of whom 146 engaged in NSSI	Verbal/emotional/physical/sexual abuse, neglect, and parental substance abuse	Participants were predominantly male (*N* = 339)	Foster placement increased the likelihood of self-harm behaviors	I = 2; II = 0; III = 0; IV = 0; V = 0; VI = 0; VII = 0. Total score = 2	Low
Swannell et al. (21)	Cross-sectional study	11,423 randomly selected adults	Emotional, physical, and sexual abuse, emotional and physical neglect	NSSI was only asked about the previous year	Physical abuse and neglect independently increased the odds of NSSI among females. Physical abuse increased the odds of NSSI among males. Sexual abuse did not independently increase the odds of NSSI for males or females. For females, self-blame had the greatest effect on the CM–NSSI relationship although dissociation and alexithymia partially mediated the relationship. For males, dissociation had the greatest effect, with self-blame also having a relatively strong effect	I = 2; II = 0; III = 0; IV = 2; V = 1; VI = 0; VII = 0. Total score = 5	Moderate
Vaughn et al. (35)	Retrospective study	45,350 adult subjects, of whom 672 engaged in NSSI. The response rate for wave I data was 81% and for wave II it was 87%, with a cumulative response rate of 70% for both waves	Sexual and physical abuse, child neglect and family violence	The study restricted analyses to adults. No validated instruments were used to assess NSSI and CM. The study did not directly evaluate the association between CM and NSSI	Less mental health and substance use comorbidity, and antisocial behavior in the low abuse/neglect class (35.7% of respondents, 91.1% male). Lower levels of antisocial behavior than the other classes were also reported in the sexual abuse class (43.1% of respondents, 98.6% female). In addition, varied and intensive forms of antisocial and externalizing behaviors emerged in the non-sexual abuse/neglect class (8.3% of respondents, 91.5% male). Moreover, high levels of clinical psychiatric and personality disorders were found in the severe high abuse/neglect/family violence class (12.95% of respondents, 100% female)	I = 2; II = 0; III = 0; IV = 0; V = 0; VI = 0; VII = 0. Total score = 2	Low
Wan et al. (34)	Retrospective study	14,221 adolescent students	Physical, emotional, and sexual abuse	The study involved only young students (10–11 years). NSSI was only asked about the previous year. Only a screening test was used to evaluate NSSI	Each type of CA was significantly associated with NSSI; a graded relation was found between the number of abusive childhood experiences and NSSI. Students who were maltreated by parents or others were at a higher risk of engaging in NSSI; the risk was greater in students maltreated by both parents and others. Students who had experienced CA with no perceived harm continued to be at an elevated risk for NSSI	I = 2; I = 0; III = 0; IV = 1; V = 0; VI = 0; VII = 0. Total score = 3	Low
Weismoore and Esposito-Smythers (28)	Cross-sectional study	263 adolescents in an acute adolescent inpatient unit	Physical and sexual abuse, physical and sexual assault	Participants were in an acute adolescent inpatient unit. Psychometric instruments used to evaluate CA and NSSI were not specific	No relationship was found between CA and NSSI. A history of assault was associated with NSSI among youths who reported higher cognitive errors and more negative self-views, even after controlling for gender and internalizing disorders	I = 2; II = 0; III = 0; IV = 1; V = 1; VI = 2; VII = 2. Total score = 8	Moderate
Zetterqvist et al. (29)	Cross-sectional study	816 adolescent students who engaged in NSSI	Physical/sexual abuse and other adverse childhood experiences	Sample likely biased toward more serious NSSI	The relation between childhood emotional, physical, and sexual abuse and carrying out NSSI for automatic reasons was mediated by symptoms of depression and dissociation. The association between physical abuse and the social functions of NSSI was mediated by symptoms of anxiety and dissociation	I = 2; II = 0; III = 0; IV = 2; V = 2; VI = 0; VII = 0. Total score = 6	Moderate
Christoffersen et al. (25)	Cross-sectional study	2,980 young subjects born in 1984 who were selected as a stratified random probability sample from a national population register of children at risk (receiving an assessment and services provided in the home)	Physical, sexual, and psychological abuse, physical neglect	The psychometric tools evaluating NSSI and CM were not specific. The sample was selected from a national population register of at-risk children	Participants with a history of child maltreatment, being bullied at school or other traumatic life events reported a rate of NSSI six times greater to that of participants without this history. The correlation between traumatic life events during adolescence and NSSI was reduced when low social support was accounted for, suggesting that social support is a partial mediator	I = 2; II = 0; III = 0; IV = 1; V = 1; VI = 0; VII = 0. Total score = 4	Moderate
Bernegger et al. (20)	Cross-sectional study	139 patients, of whom 91 (33 males and 58 females) with self-harm; 48 (13 males and 35 females) with NSSI	Physical, sexual, and emotional abuse, physical and emotional neglect	The sample was a mixed sample of patients (both inpatients and outpatients with unipolar/bipolar affective disorder). Suicidal behavior (including history of suicide attempts) as well as self-harm or NSSI were investigated. The clinical group included only patients with major depression. Only few males with CM were included	Childhood sexual abuse was a risk factor for suicide attempts but not for self-harm in adulthood. Females with a history of self-harming behavior (including suicidal intention) and NSSI had significantly higher CTQ total scores when compared with HC	I = 2; II = 0; III = 0; IV = 2; V = 2; VI = 0; VII = 0. Total score = 6	Moderate
Weierich and Nock (22)	Cross-sectional study	94 adolescents from the community	Physical/emotional/sexual abuse, physical and emotional neglect	All participants were adolescents who consented to participate in a lab-based study	Childhood sexual abuse was associated with NSSI during adolescence. Non-sexual abuse was not significantly associated with the presence/frequency of NSSI	I = 1; II = 0; III = 0; IV = 2; V = 2; VI = 0; VII = 0. Total score = 5	Moderate
Thomassin et al. (23)	Cross-sectional study	95 youths receiving inpatient psychiatric treatment	Physical, emotional, sexual abuse, physical/emotional neglect	Participants were recruited from a general psychiatric hospital.	Sexual/emotional abuse was positively correlated with NSSI. Physical abuse was not correlated with NSSI	I = 1; II = 0; III = 0; IV = 2; V = 2; VI = 0; VII = 0. Total score = 5	Moderate
Kaess et al. (18)	Cross-sectional study	125 inpatients (aged 13–26 years)	Physical abuse, sexual abuse, and neglect	The sample included only inpatients, and both children and adults. The potential risk of recall bias in association with acute mental states may have impacted on the main results. NSSI was asked only about the previous year. Control variables (e.g., depressive symptoms) were not taken into account	A history of ACE (specifically maternal antipathy and neglect) was significantly more common in patients with NSSI compared with their clinical controls. The independent and important role of childhood sexual abuse was confirmed	I = 2; II = 0; III = 0; IV = 2; V = 2; VI = 0; VII = 1. Total score = 7	Moderate
Paivio and McCulloch (17)	Cross-sectional study	100 female undergraduate students	Emotional, physical, sexual abuse, emotional, and physical neglect	The sample included only female subjects recruited in university psychology classes. All measures were self-reported	Severity of all types of maltreatment predicted a greater extent of self-injurious behavior	I = 1; II = 0; III = 0; IV = 2; V = 2; VI = 0; VII = 2. Total score = 7	Moderate

*ACE, adverse childhood experiences; BPD, borderline personality disorder; CA, child abuse; CM, childhood maltreatment; CTQ, Child Trauma Questionnaire; HC, healthy control; MDD, major depressive disorder; NSSI, non-suicidal self-injury*.

### Summary Measures

The quality of the 26 studies eventually used for this review was evaluated using the following criteria: (1) representativeness of the sample (0–2 points); (2) presence and representativeness of control group (0–2 points); (3) presence of follow-up (0–2 points); (4) evidence-based measures of CM [e.g., Childhood Experiences of Care and Abuse Interview, Child Trauma Questionnaire (CTQ), Childhood Maltreatment Interview Schedule-Short Form, Comprehensive Trauma Interview, Christchurch Trauma Assessment, Abuse and Perpetration Inventory, Childhood Trauma Interview, Comprehensive Childhood Maltreatment Scale, Traumatic Experiences Checklist, or other psychometric evaluation (0–2 points)]; (5) evidence-based measures of NSSI [e.g., NSSI Checklist, Self-Injurious Thoughts and Behaviors Interview, Deliberate Self-Harm Inventory, Comprehensive Trauma Interview, Lifetime Parasuicidal Count, Suicidal Behaviour Questionnaire, Viennese Suicide Risk Assessment Scale, Ottawa Self-Injury Inventory, Self-Injury Questionnaire, Self-Injurious Behavior Questionnaire, or other psychometric evaluation (0–2 points)]; (6) presence of raters who identified independently the presence of CM (0–2 points); and (7) statistical evaluation of interrater reliability (0–2 points).

Quality scores ranged from 0 to 14. Studies were differentiated according to their quality, as follows: (1) good quality (10–14 points), if most or all the criteria were fulfilled or, where they were not met, the study conclusions were deemed very robust; (2) moderate quality (4–9 points), if some criteria were fulfilled or, where they were not met, the study conclusions were deemed robust; and (3) low quality (0–3 points), where few criteria were fulfilled or the conclusions were not deemed robust.

## Results

### Study Sample

**Figure 1:**
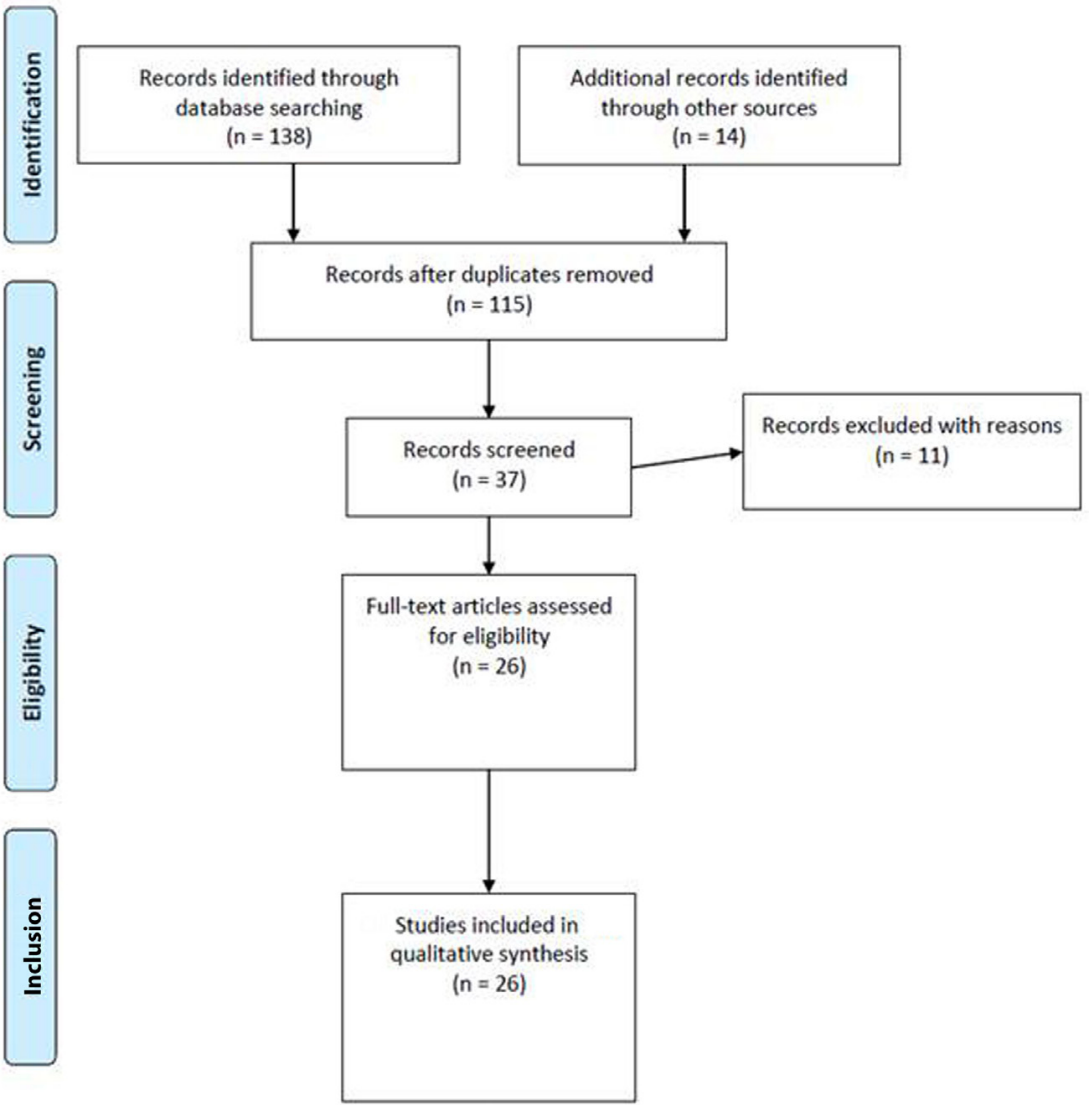
Study selection flowchart.

The searches in PubMed, Scopus, Science Direct, and PsycInfo revealed, after the removal of duplicates, a total of 26 potentially relevant articles about CM and NSSI. Overall, the search in PubMed generated 34 articles for maltreatment, 32 for abuse, and 33 for neglect; the search in Scopus generated 7 articles for maltreatment, 19 for abuse, and 2 for neglect; the search in Science Direct generated 5 articles for maltreatment, 4 for abuse, and 2 for neglect, whereas the search in PsycInfo did not provide any article. Moreover, we extracted another 14 studies from the reference lists of these articles. Of all these, 115 were excluded because they were duplicates, or they were without an abstract, or they had an abstract that did not explicitly mention NSSI and a form of CM, or they were not written in English. Then, 11 articles were excluded because they were on self-mutilation or self-harm and did not mention NSSI. Thus, 26 met our inclusion criteria and were therefore used for the present review. Figure 1 summarizes the main results of the search strategy (i.e., the identification, screening, eligibility, and inclusion process) used for selecting studies.

### Study Types and Sample Characteristics

Overall, 20 cross-sectional studies—including a total of 22,517 individuals—3 longitudinal follow-up studies—including 1,728 individuals—and 3 retrospective studies—including 62,089 individuals—were considered. Clinical samples included predominantly patients with NSSI and one of the following psychiatric diagnoses: major depressive disorder (MDD), bipolar disorder, substance use disorder, eating disorders, personality disorders or other psychiatric disorders.

### Study Quality Assessment

According to our quality score system, the mean quality score of the 20 cross-sectional studies was 5; the mean score of the 3 longitudinal studies was 6.3; and the mean score of the 3 retrospective studies was 2.3. Most of the studies (*N* = 19) were of moderate quality, one was of good quality, and six of low quality. The most relevant findings derived from the 26 studies we used are reported below.

### Cross-Sectional Studies on the Association between CM and NSSI

Most of the studies (*N* = 20) were cross-sectional in nature. Overall, only one study (quality score 10) was considered good quality, 16 studies were of moderate quality (mean quality score 5.6), and three studies were of low quality (mean quality score 2.3). All documented a positive association between NSSI and CM.

In the study of Martin and colleagues (4), NSSI thoughts were distinguished from NSSI actions, but both NSSI thoughts and NSSI actions were correlated with physical abuse. Paivio and McCulloch (17) highlighted the importance of severity of CM for NSSI. Kaess et al. (18) showed that a history of adverse childhood experiences (in particular maternal antipathy/neglect, and sexual abuse) was significantly more common in patients with NSSI than in those without. Similar results were documented by Martin and colleagues in a more recent study (19) in which NSSI behavior was linked with perceived CM. Bernegger et al. (20) analyzed a sample of adults with unipolar or bipolar affective disorder and found that childhood sexual abuse was a risk factor for suicide attempts but not for self-harm in adulthood. In that study, females who engaged in self-harming behavior (including suicidal intention) or NSSI had significantly higher CTQ scores than controls. Small-to-medium associations between specific forms of CM (in particular, sexual abuse) and the presence of a recent NSSI were also found in the study of Glassman and colleagues (12). Conversely, Swannell et al. (21) did not find evidence for the predictive role of sexual abuse for NSSI. In the study of Weierich and Nock (22), only childhood sexual abuse was positively associated with NSSI during adolescence.

On the other hand, Thomassin and colleagues (23) found that sexual and emotional abuse—but not physical abuse—were both positively correlated with NSSI. More recently, Kara et al. (24) reported that, in their sample from a forensic adolescent population, those who engaged in NSSI showed higher rates of sexual abuse and were more frequently involved in multiple crimes. In a large (*N* = 2,980) study carried out by Christoffersen and colleagues in 2015 (25), individuals with a history of CM, peer victimization at school, or other adverse experiences were six times more likely to report NSSI than participants without this history. Stewart et al. (26) confirmed that sexual abuse was associated with NSSI in a sample of adolescents who accessed mental health services. Finally, in the study of Stinson and colleagues (27) in a sample of 381 forensic mental health inpatients, traumatic experiences enhanced the likelihood of self-harm and suicide attempts.

### Studies Demonstrating the Existence of Mediators/Moderators in the Association between CM and NSSI

Overall, six studies [of which one was of good quality (quality score 10), four of moderate quality (mean quality score 6.25), and one of low quality (quality score 3)] did not find a direct correlation between CM and NSSI. In the study by Weismoore and Esposito-Smythers (28), there was no relation between childhood abuse and NSSI, but the association between assault and NSSI was significant in youth who reported more cognitive errors and more negative self-views. The retrospective study of Zetterqvist and colleagues (29) with 816 adolescents found an indirect association (mediated by depressive symptoms and dissociation) between childhood emotional or physical/sexual abuse and NSSI (see Table 1). Reichl et al. (30) showed that childhood adversity was associated with a steeper diurnal cortisol slope in the NSSI group and a flattened slope in the healthy control group. Analyzing a cohort of 422 young adult females with eating disorders, Muehlenkamp et al. (31) reported that CM likely shows an indirect association with self-harm behavior through low self-esteem, psychopathology, body dissatisfaction, and dissociation symptoms. Shenk and colleagues (32) reported that there was a significant association between CM and NSSI in females, but this relation was mediated by post-traumatic stress symptoms. Stewart et al. (2) also reported indirect links (between NSSI and physical abuse).

### Studies Not Demonstrating the Existence of an Association between CM and NSSI

Only the study of Johnstone and colleagues (5), conducted in a sample of adult outpatients with MDD, did not demonstrate the existence of a correlation between CM and NSSI. In that study, abuse was not significantly associated with NSSI or suicide attempts. The authors, however, reported a correlation between low maternal care and suicide attempts and between low paternal care and NSSI.

### Longitudinal Follow-up Studies on the Association between CM and NSSI

Overall, three studies [all of moderate quality (mean quality score 6.3)] prospectively investigated the association between CM and NSSI. Kaplan and colleagues (33) reported that abused individuals were at increased risk of NSSI at baseline. Isohookana and colleagues (13) found, in their study with 508 adolescent inpatients, that maltreatment, and specifically sexual abuse, was associated with an increased risk of NSSI and suicide attempts, although only in females. Finally, Garisch and Wilson (6) conducted a prospective study of 1,162 adolescent students and confirmed that NSSI was associated with abuse history.

### Retrospective Studies on the Association between CM and NSSI

Three retrospective studies [all of low quality (mean quality score 2.3)] analyzed the direct association between CM and NSSI. Wan and colleagues (34) demonstrated that a continuous experience of abuse was significantly associated with NSSI, independently of gender. More recently, Guvendeger Doksat et al. (14) found that a history of neglect significantly increased the risk of NSSI, whereas physical/sexual abuse increased the odds of suicide attempts. Finally, Vaughn et al. (35) found that, in a nationally representative sample including subjects who engaged in NSSI, the severity of CM was related to clinical psychiatric and personality disorders.

## Discussion and Conclusion

### Summary of Main Findings

This review was carried out to explore the association between CM and NSSI in adolescence and early adulthood. We reviewed 26 studies that investigated the association between CM and NSSI. Generally, it appears that experience of CM can increase the risk of adverse outcomes later on, in line with much evidence not reviewed here (36, 37). In addition, sexual abuse in childhood and other factors such as emotional inexpressivity, and affect intensity/reactivity can be particularly significant for both suicidal behaviors and NSSI, in line with previous findings (38–43). Only one study (5) reviewed here did not find a correlation between CM and NSSI.

There were some noteworthy gender differences. Generally, when compared to males, females who experienced childhood traumatic experiences (particularly sexual abuse) (13, 21) were more vulnerable to NSSI and suicidal behaviors. However, when physical and sexual abuse co-occurred (37) or when the experience of abuse was continuous (14), both males and females were at risk of NSSI and suicidal behaviors.

Some of the studies reviewed also tested or suggested potential mechanisms underlying the association between NSSI and CM, such as *via* post-traumatic stress disorder (22), depression (44), and emotion dysregulation (45). For instance, Weierich and Nock (22) found that re-experiencing and avoidance symptoms can mediate the relation between childhood sexual abuse and self-injury, even when controlling for major depression. Glassman et al. (12) suggested that self-criticism and cognitive dysregulation could mediate the relation between emotional abuse and self-injury, in line with much evidence that cognitive styles can be directly related to NSSI and suicidal behaviors (46, 47). The association between CM and NSSI was also mediated by post-traumatic stress symptoms in that of Shenk and colleagues (32), and low self-esteem, psychopathology, body dissatisfaction, and dissociation symptoms in that of Muehlenkamp et al. (31). These findings are in line with the suggestion that suicidal behaviors and NSSI may represent attempts to cope with dissociative symptoms or to reach a dissociative state (1, 48–50). For example, a recent meta-analysis (49) on the link between dissociation and NSSI/suicide attempts showed that those with dissociative disorders were more likely to report both previous suicide attempts and NSSI, compared to those without.

The association between CM and NSSI or suicidal behaviors was not confirmed in all studies as already suggested in the meta-analysis of Klonsky and Moyer (51). The absence of a direct association between CM and NSSI may be explained by the existence of mediators, as mentioned above. Some studies (5, 19) also suggest that high-risk family contexts rather than the experience of maltreatment may play a significant role in suicidal behaviors and NSSI.

In conclusion, therefore, CM and childhood traumatic experiences appear to increase the odds of NSSI and suicidal behaviors either directly or *via* other risk factors.

### Main Strengths and Limitations/Shortcomings

To the best of our knowledge, this is the first review to systematically evaluate the relation between CM and NSSI. However, our findings should be considered in the light of several limitations. First, we could not carry out a meta-analysis because the studies identified measured CM and NSSI differently. Importantly, we selected studies that did not exclude self-harm, suicide attempts, and other suicidal behaviors; this may have introduced confounding. In addition, although this review aimed to summarize the most relevant studies on the topic, the inclusion/exclusion of specific studies may reflect our individual point of view or expertise and training. Moreover, some studies included may have been underpowered (some had only small sample sizes and small numbers of subjects who engaged in NSSI). Another limitation is that findings may have been hampered by recall bias. In addition, the severity, duration, intensity, and age at occurrence of CM were not taken into account in all studies; these aspects of CM, potentially very important, should perhaps be considered routinely in future studies. Finally, some of the included studies were heterogeneous and/or did not include control groups.

### Implications and Future Directions

In conclusion, the findings of this systematic review support the positive association between CM and NSSI. This information may help to early detect and rapidly recognize those who experienced CM as a specific group at risk for NSSI and suicidal behaviors. Future research should explore systematically the role of vulnerability and protective factors, i.e., factors that may act to increase or attenuate, respectively, the association between CM and NSSI.

## Author Contributions

Each author consistently contributed to the paper. GS discussed and conceived the study hypothesis and wrote the main body of the paper. DE, GC, and BP performed the methodological search on the research topic and provided help in selecting papers on the main topic. GA, JN, and MBM contributed to reviewing the literature. MP, AF, and EF reviewed the paper adding contributions to the applied methodology and the discussion section. MA provided the intellectual impetuous and supervised the writing of the manuscript.

## Conflict of Interest Statement

The authors declare that the research was conducted in the absence of any commercial or financial relationships that could be construed as a potential conflict of interest.
